# Hydrophilic Versus Hydrophobic Coupling in the Pressure Dependence of the Chemical Potential of Alkali Metal and Halide Ions in Water

**DOI:** 10.1021/acs.jpcb.2c02373

**Published:** 2022-11-03

**Authors:** Luca Tonti, Franca Maria Floris

**Affiliations:** † Department of Chemical Engineering, The University of Manchester, M13 9PLManchester, U.K.; ‡ Dipartimento di Chimica e Chimica Industriale, Università di Pisa, Via Giuseppe Moruzzi 13, 56124Pisa, Italy

## Abstract

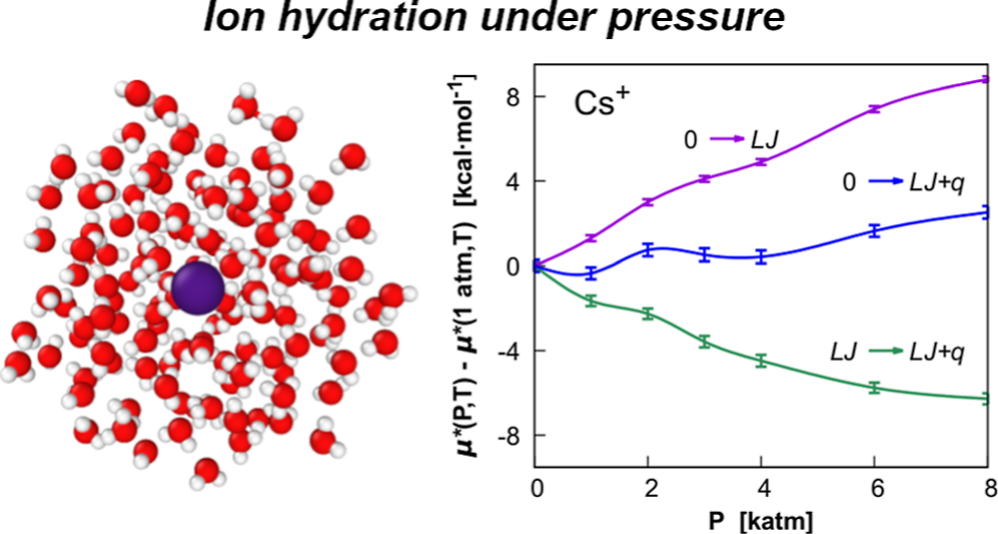

We computed the chemical potential for some alkali metal ions (K^+^, Rb^+^, and Cs^+^) and two halide ions (Br^–^ and I^–^) in aqueous solution at ambient *T* and various pressures in the range 1–8000 atm. Results were obtained from classic Monte Carlo simulations in the *NPT* ensemble by means of the free energy perturbation method. Here, the chemical potential is computed as the sum of a term relative to a Lennard-Jones solute and a term relative to the process in which this solute is transformed into the ion. Hydrophobic and hydrophilic features of these two components of the chemical potential show opposite behaviors under isothermal compression. The increase in pressure determines an increase in the hydrophobic component, which becomes more positive with a stronger effect for larger ions. Correspondingly, the values of the hydrophilic component become more negative for alkali ions, whereas they are only slightly affected by compression for halide ions. Hydrophobic–hydrophilic quasi-compensation in the slopes is observed for Rb^+^. For a smaller ion, such as K^+^, the dependence on pressure of the hydrophilic component is slightly dominant. For a larger ion, as observed in the cases of Cs^+^, Br^–^, and I^–^, the hydrophobic component assumes the determinant role. Pressure dependence of the chemical potential is little affected by corrections introduced for molecular potential truncation. This view can change for possible boundary artifacts that could have affected the static electrostatic potential. Some inference is obtained from comparison with experimental data at 1 atm on the free energy of hydration. Discrepancies show the characteristic asymmetry between cations and anions. The further addition of a correction based on the static potential significantly reduces these discrepancies with important error cancellation on the sum of chemical potentials of ions of opposite charge. The correction is applied also at higher pressures, and results are compared with those obtained by adding an alternative correction that is based on the water number density. Regardless of the ion, changes of the chemical potential induced by an increase in pressure appear to be dominated by the hydrophobic component, in particular when using the alternative correction. For bromide and iodide electrolytes, the two corrections give chemical potentials in good agreement.

## Introduction

Water is a polar solvent in which electrolytes are partially or completely dissociated into cations and anions. Specific hydration^1−3^ is shown even by monoatomic ions depending on their charge and size,^4^ as is well depicted by alkali metal and halide ions.^3,5−7^ Some of them are essential constituents of human beings and animals, while in general, they can be involved in osmosis,^8^ which is an important process occurring in biological systems. However, as the monoatomic ions are components of natural waters, their aqueous solutions at different conditions of pressure and temperature are of interest in chemistry and geochemistry.^9^

Therefore, the chemical potential of a single ion in an aqueous solution is a key molar quantity of interest in the study of a process that takes place in water or that involves the aqueous solution in a state of the process. This is because the variation of free energy in a process can be written in terms of the chemical potentials of the chemical species involved in it. The chemical potential of an electrolyte is conveniently written in terms of the chemical potential of single ions, just as the free energy, the enthalpy, and the entropy of hydration are decomposed into single-ion contributions. However, an ion cannot be added to water without adding its counterion, and thus all single-ion quantities mentioned above are not measurable but are established by convention. For instance, conventional experimental data for the free energy of hydration are obtained by the assumption that Δ_hyd_*G*° = 0 for H^+^. With regard to computed data, these are preferably obtained for the single ion. In particular, the computed value for H^+^ is used as the reference for the absolute experimental data.^1,10−12^

The free energy of hydration is a very important quantity that has been computed both within implicit models of the solvent (continuum models)^13,14^ and within methods employed in simulations.^6,15,16^ Problems in calculations are inherent to the specific method and in models used to describe the system.^16,17^ Because of the specific hydration of monoatomic ions, the basic continuum models require specific radii not only in the application of the Born formula but even in some nonhomogeneous dielectric models.^1^ On the other hand, in simulations, the truncation of the ion–water and water–water interactions requires corrections to be introduced in order to reduce discrepancies with respect to experimental data.^17^ Although these corrections^5,7,17^ can be properly managed, some aspects of the issue still remain unclear. This becomes less clear in the lack of experimental data,^1,10−12^ which are usually obtained at ambient conditions. In this context, our work aims to provide some useful discussion on simulation results obtained at higher pressures.

Following Ben-Naim, the hydration process concerns the transfer of a solute particle from a fixed position in the ideal gas to a fixed position in water at infinite dilution.^18^ With this definition, we do not need to compute the translational contribution. Furthermore, the choice of the same standard state (*C* = 1 mol/1 L) for the solute in the gas phase and in the liquid solution is particularly convenient and is usually adopted in calculations of the free energy of solvation. Since the reference states are the ideal gas in the gas phase and the solution at infinite dilution in the liquid phase, the coupling of the solute–solvent interactions plays a prominent role. This holds whether we use an implicit model^13^ of a solvent or explicit solvent molecules as in simulations.^6,16,17,19^ In the latter case, the system is usually described by effective model potential interactions, and in practice, the variation of free energy of solvation is computed from the coupling of solute–solvent interactions in the liquid phase. The internal motion contributions are often neglected or are null,^20^ as for the simple solutes studied in this work. Thus, for the hydration of a monoatomic solute M at infinite dilution, we can write
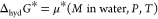
1where μ* is the chemical potential of the solute *M* in the aqueous solution at the conditions of *P* and *T*. Within a theoretical approach, it can be quite usual to decompose the hydration free energy into contributions related to solute–solvent interactions of a specific nature. This is essentially done when working with polarizable continuum models^13^ (PCMs), but it can be used even when using a discrete description of the solvent molecules.^6^ Short-range interactions, such as repulsion, dispersion, and the solute polarization under the field of water solvent, are always present,^21,22^ regardless of the solute. For a hydrophobic solute, these kind of interactions are dominant and yield unfavorable or slightly favorable Δ_hyd_G*.^20,23−25^ Charged and polar solutes are generally hydrophilic with negative values of Δ_hyd_G* due to the dominance of water polarization under the solute electric field.

In this work, the ion interacts with water by means of a Lennard-Jones (LJ) potential plus a Coulomb interaction potential.^6^ The first one provides typically a quite realistic model for a hydrophobic solute. The second is instead essential to model a charged solute, which has the characteristic of being hydrophilic. We are interested in the corresponding contributions to μ* and in their variation under the isothermal compression of the solution. We study the effect of an isothermal increase in pressure on the chemical potential of some alkali metal and halide ions in water at 298.15 K.

Despite electrostriction,^1,3,26^ for these monoatomic monovalent ions, generally a positive value of excess volumes is found from measurements at ambient conditions. The decrease in electrostriction, which was observed under isothermal compression,^3^ can indicate that the excess volumes of these ions maintain a positive sign at a higher pressure. The same can be inferred from the increase in the partial molar volume of electrolytes, which has been considered to be the main effect of pressure, albeit on the basis of few experimental data.^27^

Thus, we can expect that μ* is generally higher at a higher pressure, with changes dominated by the hydrophobic component. This is deduced from the approximate decomposition of μ* into the sum of the work of cavity formation and the Born formula when the pressure effect on cavity radii is neglected. Indeed, with these assumptions, the effect of increasing pressure on the two components is only determined by the increase in the number density and dielectric permittivity of water. This simple view does not take into account the pressure-induced effect on μ* due to changes in the hydration shells of the ion, while simulations provide valuable tools for a refined analysis. However, this implies that the problems due to the long-range nature of interactions in the aqueous solutions of ions must be addressed.

Hence, we computed the variation of free energy related to the decoupling of the ion–water interaction within the free energy perturbation^19,28,29^ (FEP) method applied to simulations at constant *P* and *T*. Having used molecular potential truncation (MPT), corrections to FEP results have been introduced. In particular, we propose a method to correct for the truncation of the water–water interactions. At 1 atm, our corrections are in line with those obtained by using more elaborated methods.^30,31^ We discuss the possible dependence on pressure of errors due to MPT in the Results and Discussion section. We also pay some attention to possible artifacts introduced by the boundary of the simulated system. This should explain the well-known asymmetry^32^ between cations and anions shown in the discrepancies with respect to experimental data^1,10,11^ at ambient conditions. We investigate how the artifact can depend on pressure by examining how the FEP results relative to the Coulomb component depend on the coupled charge. Two different methods are proposed to compute additional correction that should improve our estimate of the chemical potential. The final sections are devoted to a comparison along the isotherm of FEP results after the introduction of these corrections.

Furthermore, we reflect on implications in the evaluation of excess volumes.

## Methods

### FEP Calculations

According to Zwanzig,^33^ the difference in free energy between two states of the system can be estimated with the following formula via the FEP method
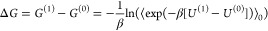
2where the superscripts (0) and (1) refer to properties of the reference and perturbed states, respectively, while angle brackets ⟨...⟩_0_ define a statistical average of the argument in the configuration space of the reference state^29^ at a fixed temperature (β ≡ 1/*kT* with *k* being the Boltzmann’s constant). When averaging in the *NVT* ensemble, the variation of the Helmholtz free energy is obtained, as in many works including the original one by Zwanzig.^33^ However, the variation of Gibbs free energy (Δ*G*) is obtained when the average is evaluated in the *NPT* ensemble.^29^

As already noted,^19^ the equation above is exact as it arises from the statistical definition of the free energy of the system.^28,33^ However, within the FEP method, the final state (1) of the process should not be too different from the initial state (0), whose assumption is necessary in practical applications in order to avoid excessively long simulations. To overcome this issue, the process, with expected Δ*G*, can be divided into a series of “windows,” whose initial and final states are fabricated intermediate states between (0) and (1) and the sum of the correspondent free energy changes is the desired change in free energy of the entire process, that is
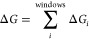
3

These intermediate states are commonly defined by a coupling parameter, λ, which scales the parameters χ of the interaction potentials
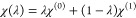
4

In this way, λ = 0 defines the initial state and λ = 1 defines the final state of the process, while the reference state of a particular window is defined in between.

In a single window, configurations are sampled for the reference potential defined by λ_*i*0_, and they are used to evaluate the energy of both the reference and perturbed states. In this work, the double-wide sampling technique^29^ was used in the computation of the free energy of a single window, whose expression is reported in eq 5
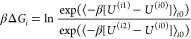
5where *i*0 is the reference state of the window, while *i*1 and *i*2 are two perturbed states whose coupling parameters differ from that of the reference state by small increments – Δλ and Δλ, respectively.^29^

In this work, the FEP method is separately applied in order to obtain Δ*G*(0 → LJ), the free energy change relative to the coupling of the ion–water LJ potential, and Δ*G*(LJ → LJ + *q*), the free energy relative to the transformation of the LJ solute into the ion. These free energy variations are summed to compute the chemical potential of the ion in aqueous solution at *P* and *T* fixed
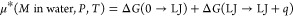
6

This decomposition of μ* is particularly convenient because in this way, we can use basic models of the two terms as a useful reference to discuss the pressure dependence of the FEP results. However, specific interest is devoted to the Coulomb term Δ*G*(LJ → LJ + *q*) because in our simulations, it is mainly subjected to errors due to MPT. Corrections to these errors can be formulated within the PCM, including Born’s formula, as shown in the next section. Hereafter, we use μ* to indicate the quantity defined in eq 6.

### Corrections for MPT

Here, we describe how we computed corrections to the FEP results in order to take into account errors due to the truncation of the ion–water and water–water interactions. We use the continuum model of the solvent, starting by computing the variation of free energy relative to the coupling of the Coulomb potential as the reversible work made to bring the ion from the vacuo in a homogeneous dielectric medium. For a monoatomic ion, the model defines a spherical cavity of appropriate radius, *R*_I_, at whose surface the relative permittivity changes its value from 1 to ϵ_r_. If, as in the simulations in this work, the ion is classically described by the field of its charge, *q*, the Born formula is obtained
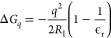
7

For a larger radius defined in terms of the ion–water cutoff radius, *R*_IW_, the Born formula can be used to compute the long-range correction to FEP results
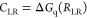
8

In this work, corrections were computed for
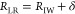
9where δ has been introduced in order to take into account the water molecule size. This seems reasonable because for the ion–water interactions, the cutoff defines the distance between the ion and the oxygen center of water molecules.

However, the cavity radius is a parameter of the continuum model, and when correcting FEP results, the most common choice corresponds to using a cavity radius exactly fixed at the ion–water cutoff. Regardless of the value fixed for this parameter in the computation of *C*_LR_, we can rewrite eq 7 as the identity
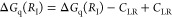
10

According to eq 10, *C*_LR_ is the proper correction to the free energy estimated as the difference between the quantities defined by eqs 7 and 8. These both arise from the integration of the same radial function, and their difference represents the contribution to the free energy provided by the dielectric contained within the spherical shell defined by *R*_I_ and *R*_LR_.

For a complete parallel with the FEP results of Δ*G*(LJ → LJ + *q*), we have introduced an error on its evaluation that is exploited to compute the correction for the truncation of water–water interactions. For this purpose, we resort to the numerical solution of the electrostatic equations which are considered in the more general applications of the continuum model. We solve the Poisson equation
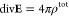
11where ρ^tot^ is the source of the field
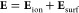
12

The total field includes that produced by σ(**r**), the charge distribution at the surface cavity due to the dielectric polarization induced by the ion field
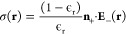
13where **n**_+_ is the unitary vector orthogonal to the surface and pointing toward the dielectric, while the electric field is evaluated inside the cavity, as indicated by the subscript. In general applications in quantum chemistry, this distribution is conveniently described by a finite number of point charges placed at the centers of the elements in which the cavity surface (Σ) has been divided. In order to solve the Poisson equation, these charges are computed after the inversion of the matrix **T**(13,34) as
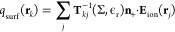
14

Finally, the contribution to the free energy of solvation is computed as half the Coulomb interaction between the polarization charges and the ion charge *q*
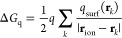
15

For a sufficient number of point charges, the equation above gives the same value as the Born formula. Nevertheless, we have introduced this formulation because it enables us to introduce an error which parallels that due to water–water cutoff in FEP calculations. Indeed, the element *kj* of the matrix **T** is used to compute the charge on **r**_*k*_, and it contains the interaction with the charge placed on **r**_*j*_ on which we can introduce a cutoff. In this way, we obtain the charges *q*_surf_^cut^ from which we obtain Δ*G*_q_^cut^. The cutoff is applied only to the larger cavity, and we compute the correction to the FEP results as
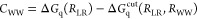
16

The calculation of Δ*G*_q_ and Δ*G*_q_^cut^ refer to a reversible charging process in which the ion charge is incremented by *dq* at each step. Instead, in the FEP calculations, the charge is incremented by a small but finite quantity, δ*q*. Therefore, the free energy is obtained by summing over the windows in which the process is divided. For a discrete charging process divided into windows, we can compute *C*_WW_ from the sum
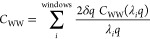
17where λ_*i*_*q* is the charge of the solute in the reference state of the corresponding window, namely, a partial sum defines the correction for a solute of charge λ_*i*_*q* + δ*q*. For the total charge *q*, using 10 windows (δ*q* = 0.05 au), eqs 16 and 17 give practically the same correction.

## Calculations

### Simulation Conditions

**Figure 1:**
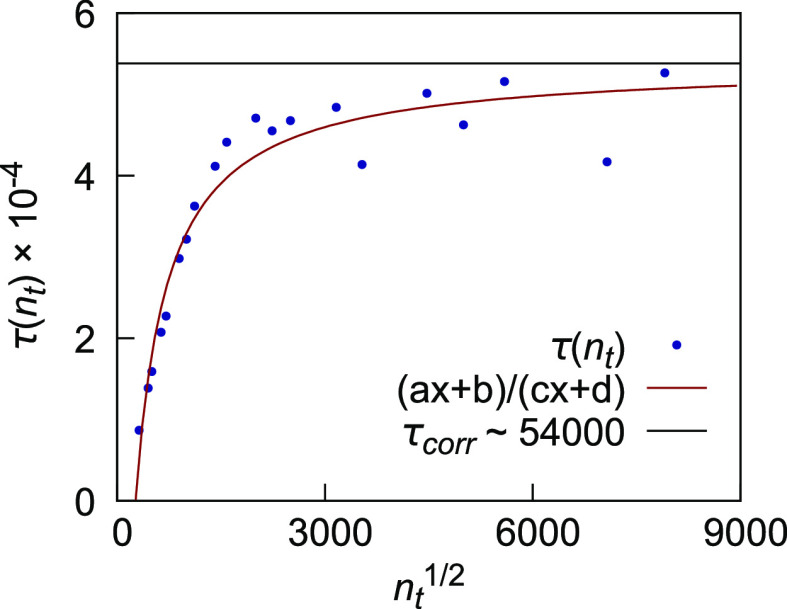
Statistical inefficiency (eq 18) plotted as a function of the square root of *n*_t_, the number of trials per block. Results were obtained from a benchmark simulation of 500 M trials for the switching off of I^–^ charge in a box of 512 TIP4P waters with periodic boundary conditions at 1 atm and ambient temperature. The correspondent free energy was computed via the FEP method for blocks of 50 K trials each. Averages of single blocks were combined to estimate σ^2^(⟨Δ*G*⟩_b_). The plateau gives an estimate of the correlation time between configurations (τ_corr_ ∼ 54,000).

In this work, we computed the chemical potential of the alkali metal ions K^+^, Rb^+^, and Cs^+^ and the halide ions Br^–^ and I^–^ at 1, 1000, 2000, 3000, 4000, 6000, and 8000 atm of pressure and ambient temperature, performing *NPT* Monte Carlo simulations of one single ion at a fixed position at the center of a cubic box surrounded by 512 water molecules with periodic boundary conditions. All simulations were run with BOSS software, version 4.9.^35^ Water–water interactions were described by the TIP4P water model,^36^ whose charges interact with the charge of the ion with a Coulomb potential. The nonelectrostatic interactions between the ion and water were described by the LJ potential with parameters derived from the LJ parameters of TIP4P water and the LJ parameters of Jensen and Jorgensen^6^ for the ion. MPTs were applied using cutoff distances of 11.5 Å for both interactions. The chemical potential of the ion was evaluated by performing FEP calculations, switching off ion–water interactions in two stages. Starting from equilibrated systems of one ion in water, whose structural analysis is already reported in our previous work,^3^ the ion charge was gradually switched off while maintaining LJ interactions active to obtain Δ*G*(LJ + *q* → LJ). This process was split into 10 windows of double-wide sampling; 10 M trials of equilibration plus 20 M trials of averaging were performed for each window. After the charge was completely switched off, we estimated Δ*G*(LJ → 0) by annihilating the ion–water LJ potential gradually in 5 windows of double-wide sampling, with 20 M trials of equilibration and 160 M trials of averaging for each. The setup for the estimation of Δ*G*(LJ → 0) was chosen in order to have higher values in magnitude of the free energy of a single window (using fewer windows) and to reduce its standard deviation (using more configurations for averaging). Values of Δ*G*_*i*_ were computed as an average of single values per block of 2 M trials. To ensure uncorrelation between block averages, we estimated the correlation time by computing the statistical inefficiency of the free energy change for the switching off of the charge of I^–^ in water at 1 atm using the definition by Friedberg and Cameron^37,38^
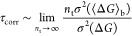
18where *n*_t_ is the number of trials per block and σ^2^(⟨Δ*G*⟩_b_) and σ^2^(Δ*G*) are the variance across blocks and the total variance of the run, respectively. Our analysis, whose results are reported in Figure 1 plotting the quantity of eq 18 as a function of , for blocks of different sizes showed that the correlation time for this specific process was ∼54,000 trials, 2 orders of magnitude smaller than the length of one block, proving that our blocks of 2 M trials each can be safely considered uncorrelated. The asymptotic value τ_corr_ is extrapolated via nonlinear regression of the dataset, as is indicated in the figure.

### Cutoff Setup and Corrections for MPT

For monoatomic ions in water, errors due to the truncation of ion–water and water–water interactions are of opposite sign, and hence they partially cancel each other.^6^ The degree of cancellation critically depends on *R*_WW_ for a fixed value of *R*_IW_. We exploit information derived from detailed studies^6^ at 1 atm and limit our tests to cutoff radii of 10 and 11.5 Å. According to the literature, the best performance in terms of convergence is reached beyond 9 Å when *R*_WW_ = *R*_IW_. This is confirmed by the Coulomb component of our FEP results, which show agreement within 2 times the statistical uncertainties for the two tested radii (see the Supporting Information). Thus, a cutoff of 11.5 Å for both interactions was adopted in our calculations.

A performance which is not as good is obtained when different cutoffs are used for water–water and ion–water interactions.^6^ For *R*_WW_ = 10 Å, the results of Δ*G*(0 → LJ) are practically converged, according to our test made for I^–^, at all values of pressure that we studied along the isotherm. This does not occur for the results of Δ*G*(LJ → LJ + *q*), which compared with the reference data (*R*_WW_ = 11.5 Å) show significant differences. These give a measure of errors caused by neglecting the interactions between waters whose oxygen centers have distances in between 10 and 11.5 Å. Reduction of their magnitude is observed by the addition of *C*_WW_ to the original FEP results (see Supporting Information). In this comparison, the addition of *C*_LR_ is irrelevant.

Of course, since both corrections (eqs 8 and 16) are formulated within the PCM of the solvent, these can only approximately correct the FEP results. We expect the model, despite describing water as a homogeneous dielectric medium, to provide the right sign and the order of magnitude of the main electrostatic errors.

**Figure 2:**
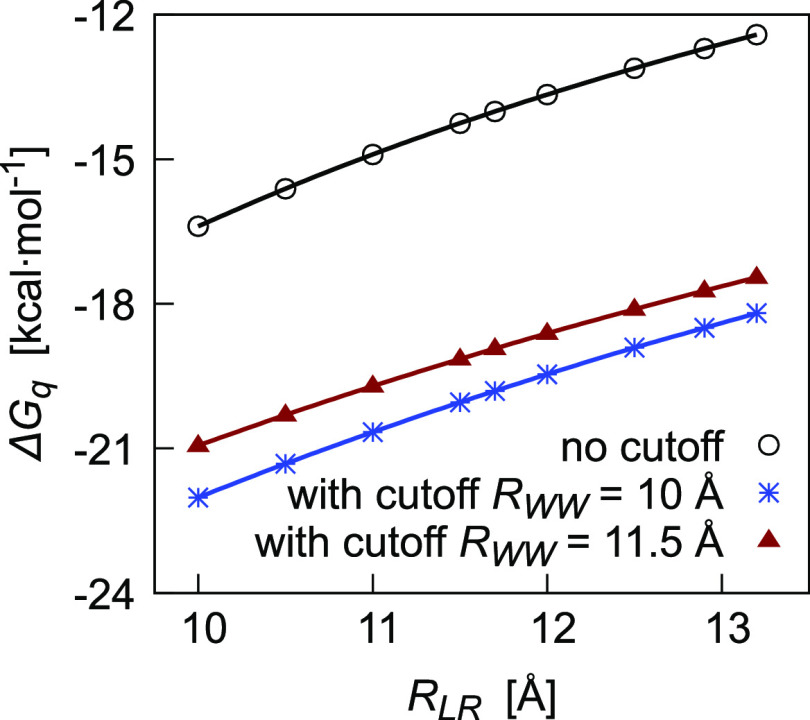
Dependence on the cavity radius of Δ*G*_q_ for an ion modeled as a point charge (*q* = 1 au) enclosed in a large cavity. Results obtained from eq 15, without using any cutoff (circles) and using the cutoff of 10 Å (stars) and of 11.5 Å (triangles) in the definition of the **T** matrix.

The correction *C*_WW_ (eq 16) is of a positive sign because the cutoff in the calculation of the matrix **T** leads to a more negative free energy (eq 15), as shown in Figure 2. Regardless of the cavity radius *R*_LR_, *C*_WW_ is smaller for the larger value of *R*_WW_ (see Table 1). This is in line with what is found in the literature with alternative methods.^30,31^ For comparable cutoff radii, our correction is in between these data. By using the same cutoff for both interactions, Wood^30^ computed a correction of 3.3 kcal/mol for a cutoff of 12 Å. On the other hand, Resat and McCammon^31^ found a correction of 7.32 kcal/mol for a cutoff of 11 Å.

**Table 1:** Dependence of *C*_WW_ (kcal/mol) on the Cavity Radius, *R*_LR_, for a Water–Water Cutoff of 10 Å (Second Column) and of 11.5 Å (Last Column) at 1 atm^a^

*R*_LR_ (Å)	*C*_WW_(10 Å)	*C*_WW_(11.5 Å)
10.0	5.63	4.55
11.5	5.79	4.89
11.7	5.80	4.92
12.9	5.79	5.02
13.2	5.78	5.03

a The number of point charges on the surface was 1000.

Finally, the whole correction *C*_LR_ + *C*_WW_ is of a negative sign and is practically independent of pressure (see Table 2). This appears as to be a consequence of the low dependence of ϵ_r_ on pressure.

**Table 2:** Pressure Dependence of Corrections Based on a Cavity Surface of 13.2 Å and 1000 Points^a^

*P* (atm)	*C*_LR_	*C*_WW_	*C*_LR_ + *C*_WW_
1	–12.42	5.03	–7.39
4000	–12.44	5.05	–7.39
8000	–12.45	5.06	–7.39

a Results for *C*_WW_ refer to a cutoff of 11.5 Å. Experimental values of the static dielectric constant of water were used.^39^

## Results and Discussion

### FEP Results: Pressure Dependence of μ* and Its Hydrophobic and Hydrophilic Components

The FEP results for alkali metal and halide ions computed at 298.15 K and various pressures from 1 up to 8000 atm are shown in Tables 3–5. Results in Tables 3 and 4 refer, respectively, to the scaling of the LJ and Coulomb terms of the ion–water interaction potential. Regardless of pressure, the corresponding changes in free energy are positive (Table 3) and negative (Table 4), and therefore they can be defined as the hydrophobic and hydrophilic components of μ* (Table 5) in accordance with eq 6. Results in Table 5 represent the FEP estimate of the chemical potential of the ion, without the addition of any corrective term. However, in this work, we have proposed how to compute corrections for errors introduced by the truncation of the Coulomb interactions. These are important when comparing experimental and simulation results of Δ_hyd_G*.^1,6,10,11^

**Table 3:** Values in kcal/mol^a^ of Δ*G*(0 → LJ) at 298.15 K and Various Pressures from 1 to 8000 atm Obtained by FEP Calculations in an *NPT* Ensemble

Δ*G*(0 → LJ)(kcal/mol)					
*P* (atm)	K^+^	Rb^+^	Cs^+^	Br^–^	I^–^
1	5.37(8)	6.25(9)	7.3(1)	0.6(1)	1.1(2)
1000	6.66(9)	7.8(1)	8.6(1)	2.2(1)	2.5(2)
2000	7.30(9)	8.4(1)	10.3(1)	3.1(1)	4.4(2)
3000	8.8(1)	9.4(1)	11.4(1)	4.0(2)	5.0(2)
4000	9.2(1)	10.5(1)	12.2(1)	4.6(2)	6.5(2)
6000	10.4(1)	12.0(1)	14.7(1)	7.2(2)	8.9(2)
8000	11.5(1)	13.4(1)	16.1(1)	9.2(2)	12.1(3)

a The numbers in parentheses are the statistical uncertainties in the last digit.

**Table 4:** Values in kcal/mol^a^ of Δ*G*(LJ → LJ + *q*) at 298.15 K and Various Pressures from 1 to 8000 atm Obtained by FEP Calculations in an *NPT* Ensemble

Δ*G*(LJ → LJ + *q*)(kcal/mol)					
*P* (atm)	K^+^	Rb^+^	Cs^+^	Br^–^	I^–^
1	–76.3(2)	–72.4(2)	–67.6(2)	–57.9(2)	–49.4(2)
1000	–77.6(2)	–73.6(2)	–69.2(2)	–57.1(3)	–49.1(3)
2000	–78.4(2)	–75.1(2)	–69.8(2)	–57.5(2)	–49.1(2)
3000	–79.9(2)	–75.9(2)	–71.2(2)	–58.0(2)	–49.2(2)
4000	–81.2(2)	–76.7(2)	–72.1(2)	–57.7(2)	–48.2(2)
6000	–81.9(2)	–77.7(2)	–73.3(2)	–57.1(2)	–48.7(2)
8000	–83.4(2)	–79.2(1)	–73.9(2)	–56.9(2)	–48.1(2)

a The numbers in parentheses are the statistical uncertainties in the last digit.

**Table 5:** FEP Values in kcal/mol^a^ of Δ*G*(0 → LJ + *q*) at 298.15 K and Various Pressures from 1 to 8000 atm Obtained as the Sum of Values in Tables 3 and in 4^b^

Δ*G*(0 → LJ + *q*)(kcal/mol)					
*P* (atm)	K^+^	Rb^+^	Cs^+^	Br^–^	I^–^
1	–70.9(2)	–66.2(2)	–60.3(2)	–57.3(2)	–48.3(3)
1000	–70.9(2)	–65.8(2)	–60.6(2)	–54.9(3)	–46.6(4)
2000	–71.1(2)	–66.7(2)	–59.5(2)	–54.4(2)	–44.7(3)
3000	–71.1(2)	–66.5(2)	–59.8(2)	–54.0(3)	–44.2(3)
4000	–72.0(2)	–66.2(2)	–59.9(2)	–53.1(3)	–41.7(3)
6000	–71.5(2)	–65.7(2)	–58.6(2)	–49.9(3)	–39.8(3)
8000	–71.9(2)	–65.8(1)	–57.8(2)	–47.7(3)	–36.0(4)

a The numbers in parentheses are the statistical uncertainties in the last digit.

b According to eq 6, data in this table correspond to the FEP estimates of μ* without any additional correction.

**Figure 3:**
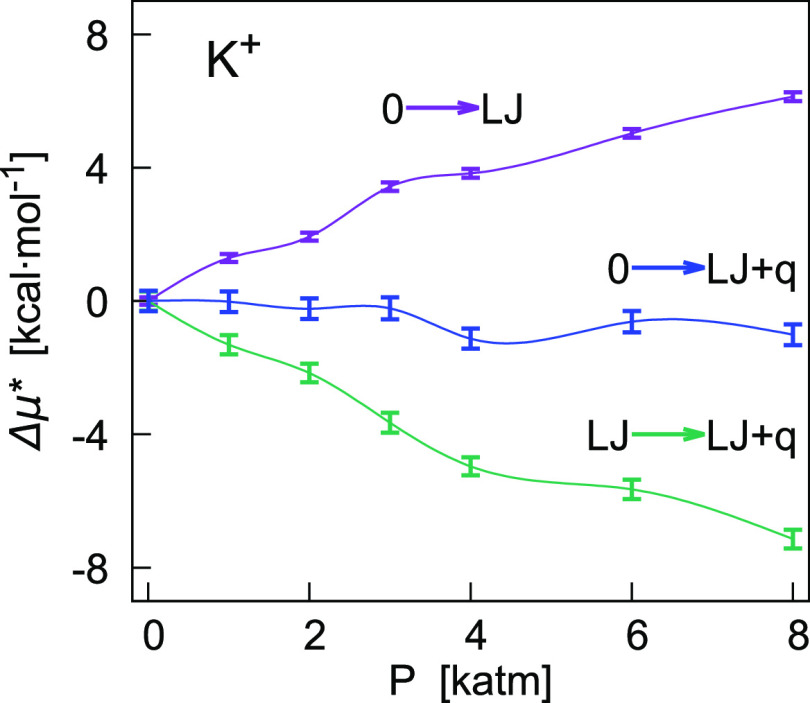
Pressure dependence of Δμ* = μ*(*P*) – μ*(1 atm) (kcal/mol) and its components (eq 6) obtained from FEP calculations for K^+^ in TIP4P water at 298.15 K. Blue points correspond to the full coupling of the ion–water potential (0 → LJ + *q*), violet points to the coupling of the its LJ term (0 → LJ), and green points are relative to the transformation of the LJ solute into the ion (LJ → LJ + *q*). The chemical potential and its Coulomb component include the corrections *C*_LR_ and *C*_WW_. Lines in the figure were drawn to guide the eye only.

**Figure 4:**
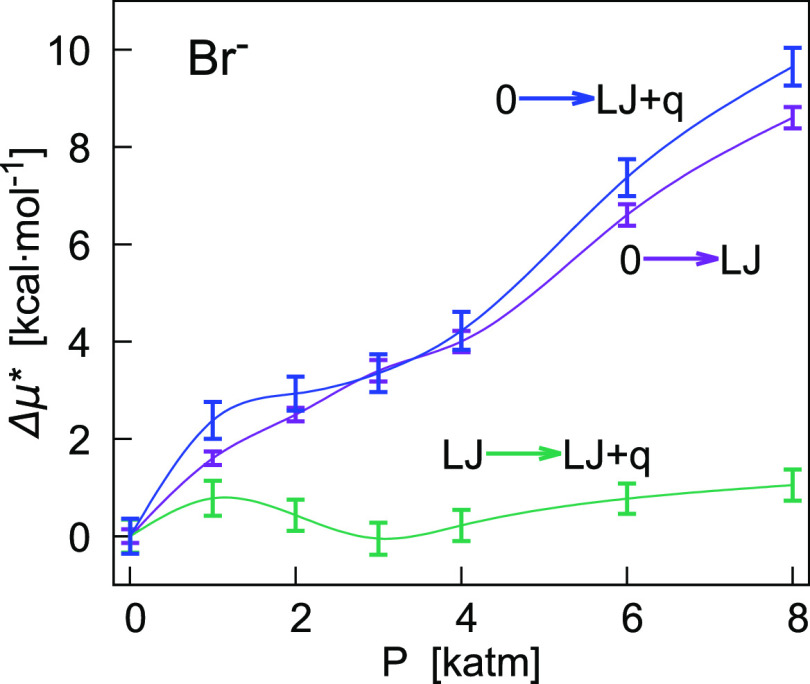
Pressure dependence of Δμ* = μ*(*P*) – μ*(1 atm) (kcal/mol) and its components (eq 6) obtained from FEP calculations for Br^–^ in TIP4P water at 298.15 K. Blue points correspond to the full coupling of the ion–water potential (0 → LJ + *q*), violet points to the coupling of the its LJ term (0 → LJ), and green points are relative to the transformation of the LJ solute into the ion (LJ → LJ + *q*). The chemical potential and its Coulomb component include the corrections *C*_LR_ and *C*_WW_. Lines in the figure were drawn to guide the eye only.

Here, we focus on the free energy changes caused by an increase in pressure of the liquid solution at ambient temperature. For K^+^ and Br^–^, Figures 3 and 4 show the difference between results at a value of *P* with respect to results at 1 atm. Differently from data in Tables 3–5, these include the above corrections, *C*_LR_ and *C*_WW_, which take into account respectively the cutoff in the ion–water and water–water Coulomb interactions. These corrections weakly depend on *P* (see Table 2) so that the original FEP results have practically the same profiles shown in the figures.

For K^+^, the pressure effect on the two components Δ*G*(0 → LJ) and Δ*G*(LJ → LJ + *q*) is equally important (Figure 3). Being of opposite sign, these two terms partially compensate up to 3000 atm, whereas the Coulomb term becomes slightly more important at higher pressures (). As shown in Table 4, at higher pressures, the Coulomb component becomes significantly more favorable to the hydration of the ion. Differently, changes of μ* with pressure for Br^–^ are predominantly affected by the LJ component, while the Coulomb component generally shows variations within 3 times the statistical uncertainty (Table 4). Nevertheless, the interpolating curves (Figure 4) seem to indicate that the coupling of the Coulomb interaction potential can affect the profile of μ* (Δ*G*(0 → LJ + *q*)).

The main features described above for K^+^ are shared by Rb^+^ and Cs^+^, as shown in Tables 3–5. The same can be said when comparing I^–^ with Br^–^. The difference in the values of μ* are shown in Table 6 with respect to K^+^ for alkali metal ions and with respect to Br^–^ for I^–^. Corrections *C*_LR_ and *C*_WW_ have no influence on these values. It is worth noting that at 1 atm, these values are in excellent agreement with the corresponding differences in Δ_hyd_G* obtained from the experimental values of the Marcus compilation^1^ (see footnote of Table 6). Thus, these data can be considered reliable also in the free energy components arising from the separate coupling of the LJ and Coulomb ion–water interactions, which are discussed in the following.

**Table 6:** Values in kcal/mol^a^ for Difference in Δ*G*(0 → LJ + *q*) between Two Ions at 298.15 K and Various Pressures from 1 to 8000 atm Obtained by FEP Calculations in the *NPT* Ensemble^b^

*P* (atm)	Rb^+^–K^+^	Cs^+^–K^+^	I^–^–Br^–^
1^a^	4.8(3)	10.6(3)	9.0(4)
1000	5.1(3)	10.3(3)	8.3(5)
2000	4.4(3)	11.6(3)	9.7(4)
3000	4.6(3)	11.3(3)	9.8(4)
4000	5.8(3)	12.1(3)	11.4(4)
6000	5.8(3)	12.9(3)	10.1(4)
8000	6.1(3)	14.1(3)	11.7(5)

a At 1 atm, experimental results for the difference in the free energy of hydration are 4.8 kcal/mol for Rb^+^–K^+^, 10.7 kcal/mol for Cs^+^–K^+^, and 9.6 kcal/mol for I^–^–Br^–^.

b This difference corresponds to the difference in μ* between two ions. The numbers in parentheses are the statistical uncertainties in the last digit.

**Figure 5:**
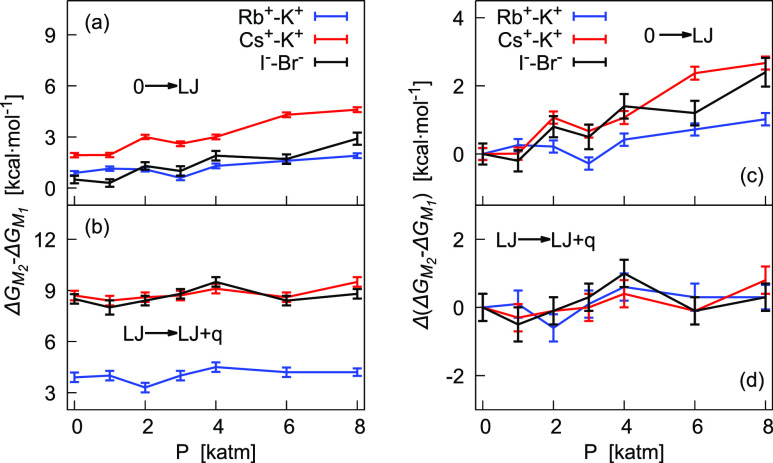
Pressure dependence at 298.15 K of the difference in μ* (kcal/mol) and its components for pairs of ions of the same charge, Rb^+^–K^+^, Cs^+^–K^+^, and I^–^–Br^–^. Panels (a,b) show the absolute values (see Table 6) with  = μ*(*M*_1_). Relative values with respect to values at 1 atm are in panels (c,d).

When comparing ions of the same charge, differences in the free energy of hydration are mainly related to a different ion size. Differences in Pauling radii^40^ are 0.15 and 0.36 Å, respectively, for Rb^+^ and Cs^+^ with respect to K^+^ and 0.21 Å for I^–^ with respect to Br^–^. Results in Table 6 show that a larger difference in free energy corresponds to a larger difference in size. This effect is enhanced at higher pressures. In our calculations, these results arise from differences in the LJ parameters and precisely only from differences in the σ parameter.^6^ The term most affected by this change is Δ*G*(LJ → LJ + *q*), as shown in panel (b) of Figure 5. However, the hydrophobic term, Δ*G*(0 → LJ), is subjected to more important changes under compression. This is better appreciated in panels (c,d) of the same figure, where we have plotted relative values with respect to those evaluated at 1 atm. The effect of compression is indeed less relevant on the Coulomb term also when comparing Cs^+^ with K^+^ [panel (d)].

**Figure 6:**
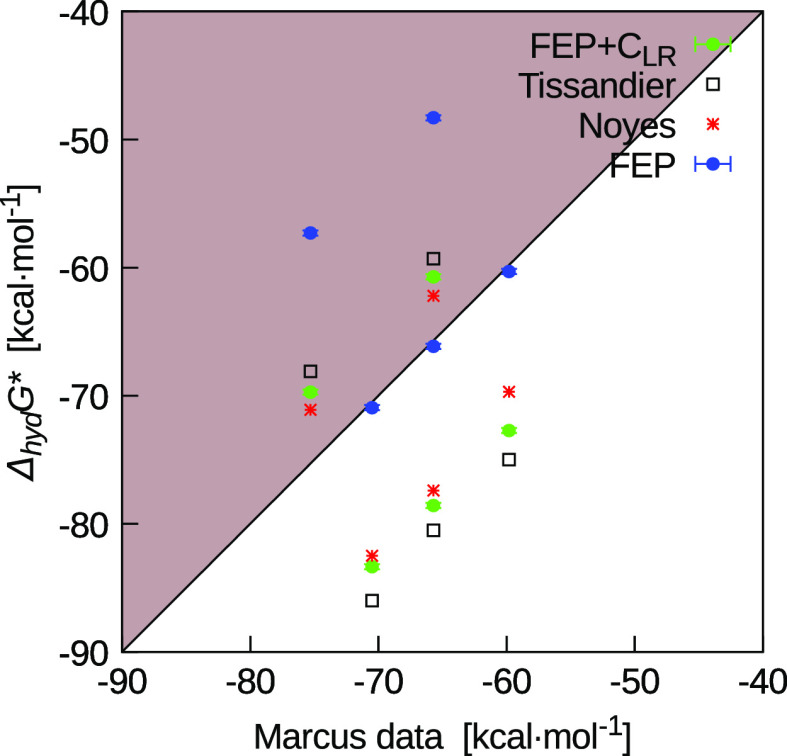
Comparison at 1 atm and 298.15 K between FEP and FEP + *C*_LR_ results with experimental data for K^+^, Rb^+^, and Cs^+^ (below the shaded area) and the halide ions, Br^–^ and I^–^ (above the shaded area). For consistent comparison, the Tissandier et al.^11^ and Noyes^10^ data were converted to the same standard state of the Marcus data,^1^ that is an ideal gas at a gas-phase concentration of 1 mol/L and an ideal solution at a liquid-phase concentration of 1 mol/L.

Discrepancies with experimental data are instead significant for the difference in free energy between ions of opposite charges. As to the original FEP results, this reflects significant discrepancies in the single-ion quantities of the anions since excellent agreement is obtained for cations (Figure 6). However, the Marcus compilation was used as target by Jensen and Jorgensen^6^ in the optimization of the ion–water model potential. Regardless of the ion charge, experimental data are well reproduced by FEP results in droplet calculations using spherical boundary conditions.^6^ Thus, the significant discrepancies observed for halide ions in this work are to be ascribed to the different simulation conditions.

### Understanding the Behavior of the Hydrophobic and Coulomb Terms

For the chemical potential of an ion dissolved in water, a complete understanding of the two components (eq 6) should require a detailed analysis which involves hydration structure as well as energy and entropy contributions.^41^ Here, we question about the reasonableness of our results on the basis of general considerations mainly related to the ion–water interaction model. We recall that the parameters used in this work were those of Jensen and Jorgensen,^6^ which were optimized on structure and free energy of hydration at ambient conditions. For these parameters as well as for the TIP4P model, we assume transferability at higher pressures.

As regards the hydrophobic component, Δ*G*(0 → LJ), its positive sign is in line with what is generally expected for a hydrophobic solute. This should occur because the work of cavity formation^42−45^ prevails over the solute–water interaction according to the most common decomposition^23,46^ of free energy adopted for these solutes and in general when working in the framework of continuum solvation theory.^13,21^ In this work, the LJ potentials of alkali metal ions are very weakly attractive, with parameter σ increasing with the ion size but with the same parameter ϵ. This explains the larger values obtained for alkali metal ions at 1 atm and opposite dependence on size with respect to the experimental data of noble gases.^47^ A similar trend was obtained by Ashbaugh and Asthagiri,^7^ although the free energies are less than half our values (Table 3). Although a different method and a different setup for simulations were used, such discrepancies should mainly reflect differences in the LJ parameters.^5^ For halide ions, discrepancies are even greater, but in this case, they obtained larger values^7^ than ours, which are closer to those of noble gases. At higher pressures, we cannot make a consistent comparison, even though we are confident enough about the higher positive values of the hydrophobic component. Indeed, this is in line with positive excess volumes obtained for aqueous solutions of methane^48^ and of hard spheres of various sizes.^49^

As observed in the previous section, the LJ parameters are fundamental to obtain a realistic comparison between ions of the same charge and therefore entail dependence on the ion size. Both the free energy components (Tables 3 and 4) are affected by changes in the σ parameter of the LJ ion-O potential. The significant effect on the Coulomb component manifests itself through specific structural changes in the hydration shells.^3,6^ This should imply a specific local dielectric answer,^1,14^ and this is one of the reasons that justifies specific cavity radii when using Born’s formula to model this term (see eq 7). The very different behavior of the Coulomb term (Table 4) shown with an increase in pressure between the alkali metal and halide ions should be related to a very different pressure effect on the structure of hydration shells.^3^ This implies that Born’s radii should have a very different dependence on pressure. Indeed, when using Born’s formula without changing the radius, the increase with pressure of dielectric permittivity of water yields only a weak monotonic decrease of the Coulomb component.

For an ion in an aqueous solution, the Coulomb component is dominant. This is generally true, though its relative importance can specifically depend on pressure. In this regard, results are affected by LJ parameters, the simulation setup, and the method used to compute free energies. For example, the Coulomb component is often not directly comparable with the electrostatic contribution to the solvation energy, although both can be modeled with Born’s formula (eq 7). This is a consequence of a different decomposition of the free energy.^50^ Indeed, the contribution due to solute polarization under the solvent field can be included in the electrostatic contribution, while in this work, it is mainly contained in the hydrophobic component due to the fitting of LJ parameters.^6^ Still, within the framework of the PCM, the electrostatic contribution includes mutual polarization, in particular when the solute is treated at a quantum mechanical level.^13,14,31,34^

In the following section, we focus on the simulation set up, which can have a significant effect on the Coulomb component. We expect that this can have some influence on the pressure profile of μ*.

### The Coulomb Component: Dependence on the Scaled Charge and Boundary Artifact

**Figure 7:**
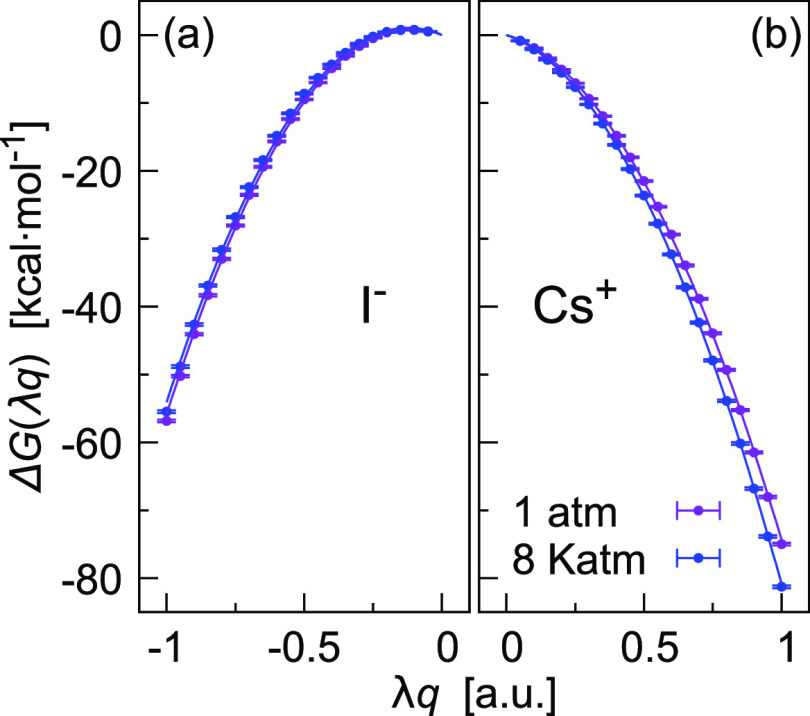
FEP results for I^–^ (left) and Cs^+^ (right) of Δ*G*(LJ → LJ + *q*) as a function of the scaled charge (λ*q*) at 1 and 8000 atm and 298.15 K. Results include the corrections *C*_LR_ and *C*_WW_. Curves obtained by fitting with eq 19.

Regardless of pressure, FEP results relative to the coupling of the ion–water Coulomb interaction potential are well fitted by the function
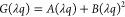
19where *q* is the ion charge and λ is the coupling parameter. For λ = 0, we have the LJ solute, and for λ = 1, the ion. Since the corrections *C*_LR_ and *C*_WW_ scale with the square of the charge, eq 19 is valid also for the FEP corrected results. Figure 7 shows these results at 1 and 8000 atm for Cs^+^ and I^–^. The fitted data include corrections properly computed at each scaled value of the charge according to eq 17. The term linear in *q* represents the interaction between the solute charge and a static electrostatic potential *A*. According to other works,^17^ we find that this interaction is attractive for cations and repulsive for anions. This term can include systematic errors due to boundary conditions in FEP calculations. Indeed, a shift in this potential explains the discrepancy between simulation results obtained with Ewald summation and those obtained by MPT.^17^

In another work, the origin of the linear term has been investigated for λ → 0, where it becomes dominant.^5,17^ At the center of the uncharged solute (*q* = 0), the first hydration shell gives a non-zero contribution due to specific water orientations.^5,17,32,42^ In spite of this, it has been shown that the final value is mainly determined by waters placed around the IW boundary cutoff.^17^ Hence, it seems that the static electrostatic potential can arise from a boundary artifact due to the truncation of the ion–water interaction potential. This yields a discontinuity at *R*_IW_ for the ion-O distribution, while the ion-H distribution is non-zero outside the cutoff sphere.^17^ When the water molecule is not simply described by a point dipole, one can compute an electrostatic potential at the center of the ion, with attractive or repulsive interaction depending on the sign of the ion charge. Ashbaugh and Wood^17^ performed the calculation for SPC/E water and obtained a value of −10 kcal/(mol e), where e is the elementary charge. Little dependence on the water model has been found for a neutral solute.^5^ Concerning our calculations, a close value should be obtained for TIP4P water using similar approximated distributions for the charge sites. These are based on the assumption that waters responsible for the boundary artifact are weakly correlated with the central solute. This may not be valid to the same extent for a ion,^17^ though for monoatomic ions, we have shown that the average water-dipole orientation is little correlated with the central ion at distances beyond the second shell.^3^

The merger between the boundary artifact with real effects not yet clearly completely recognized is also possible. From fitting in limited intervals of λ, we confine ourselves to observing that the parameter *A* is very well defined in [0:0.2] but less well defined in [0.7:1]. For λ → 0, the ion field is very weak, and also waters in the first hydration shells should have contributed to the potential.^17,32^ In this case, limitations of Born’s formula are possible^5^ and an extended continuum model theory should be necessary to include appropriate corrections.^13,17,51^ At the same time, in FEP calculations, specific meaning has been ascribed to the linear term evaluated in the limit of λ approaching zero.^5,52^ In the opposite limit, for λ → 1, where the ion field is stronger, the less good definition of *A* for λ in [0.7:1] could also arise from some inadequacy of the fitting function.^5^

Thus, one could make the assumption that the boundary artifact is much more reflected in *A* when fitting in the intermediate interval.

**Figure 8:**
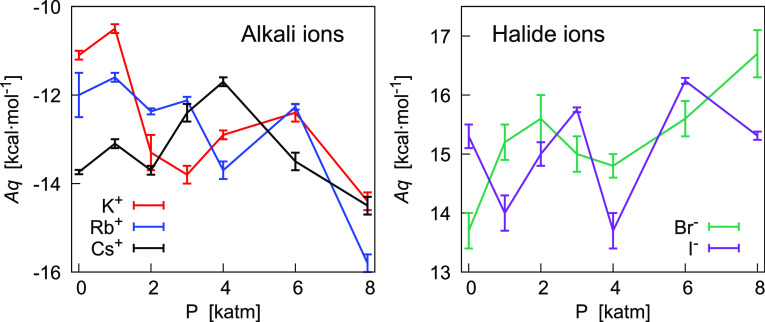
Pressure dependence of *Aq* for the cations K^+^, Rb^+^, and Cs^+^ (left) and the anions Br^–^ and I^–^ (right). The static potential *A* was obtained from the fitting with eq 19 in [0.2:0.5] of Δ*G*(LJ → LJ + *q*) as a function of λ*q* at constant *P* and *T*.

The corresponding interaction energy with the ion (*Aq*) is plotted against pressure in Figure 8. Over the entire range, variations are within 3–4 kcal/mol, although in many cases, differences between values at two different values of *P* are not very significant. Statistical uncertainties coupled with the fluctuating behavior of the data do not allow us to achieve conclusions concerning the possible dependence on pressure of *Aq*. At the same time, the trend of fluctuations appears to depend on the ion, even though at various pressures, comparison between ions shows that values can be very close within a few times the statistical uncertainty. This can depend on the range of fitted data. For example, at 1 atm, values are well separated when fitting in [0.2–0.5] (Figure 8). Instead, for λ within 0.2, we found the same value of *A* for alkali metal ions (≃−13.2 kcal/mol), a value of −11.7 kcal/mol for Br^–^ and of −13.5 kcal/mol for I^–^.

### Comparison with Experimental Results

First of all, we make a comparison at ambient conditions with the data used as the target in modeling the LJ parameters of the ions.^6^ Discrepancies between our FEP results and the Marcus compilation^1^ of experimental hydration free energies are shown in Table 7. Values of δΔ*G*_1_ refer to the uncorrected results, while values of δΔ*G*_2_ and δΔ*G*_3_ refer to results corrected by *C*_LR_ and by *C*_LR_ + *C*_WW_, respectively.

**Table 7:** Experimental Free Energies of Hydration from the Marcus Compilation, which were Used as a Target by Jensen and Jorgensen, and Discrepancies from These Data for Simulation FEP Results of This Work (δΔ*G*_1_)^a^

ion	Δ*G*_hyd_^exp^	δΔ*G*_1_	δΔ*G*_2_	δΔ*G*_3_	δΔ*G*_3_ – *Aq*	δΔ*G*_3_ – Φ*q*
K^+^	–70.5	–0.4	–12.8	–7.8	3.3	6.7
Rb^+^	–65.7	–0.5	–12.9	–7.9	4.1	6.6
Cs^+^	–59.8	–0.5	–12.9	–7.9	5.8	6.6
Br^–^	–75.3	18.0	5.6	10.6	–3.1	–3.9
I^–^	–65.7	17.4	5.0	10.0	–5.3	–4.5

a Values of δΔ*G*_2_ and δΔ*G*_3_ refer, respectively, to discrepancies after corrections *C*_LR_ and *C*_LR_ + *C*_WW_ are added to the original FEP results.

Without using any correction, results for alkali metal ions show very good agreement, while results for halide ions are less negative by 17–18 kcal/mol. The addition of *C*_LR_ to the FEP results causes for cations’ negative deviations of the free energy from reference values,^1^ with δΔ*G*_2_ up to −12.9 kcal/mol, while it gives more correct results for anions, with deviations decreasing from δΔ*G*_1_ = 17.4–18 to δΔ*G*_2_ = 5–5.6 kcal/mol. With this correction, there is a partial error compensation on the sum of the free energies of an alkali metal ion and a halide ion. This is important since the sum defines the hydration free energy of the corresponding electrolyte, the quantity which is experimentally determined. When using the original FEP results, we obtained values of the sum that are less negative by 17–17.5 kcal/mol. These become more negative by 7.3–7.9 kcal/mol when the corrected results are used. Further compensation is obtained when adding the correction *C*_WW_, with an error of 2.1–2.8 kcal/mol in the sum. Regarding the difference between the free energies of two ions, corrected and uncorrected results show the same features, with good or poor agreement with reference values^1^ for differences between ions of the same or opposite charge, respectively (see also Table 6 and Figure 5).

Turning to the results of single ions, the sign of δΔ*G*_3_ is in line with the error’s sign due to the possible boundary artifact discussed in the previous subsection. Assuming that *Aq* estimates this error, improved agreement with the Marcus compilation is recovered. The corresponding discrepancy, δΔ*G*_3_ – *Aq*, is shown in Table 7 for *A* determined by the fitting with eq 19 in [0.2:0.5]. With this correction, there is a perfect compensation of errors when computing the hydration free energy for KBr and CsI. Discrepancies are within 2.5 kcal/mol for the other possible electrolytes. Regarding differences between ions of the same charge, the maximum discrepancy in modulus is 2.7 kcal/mol, while for ions of opposite charge, this is 11.6 kcal/mol.

Finally, δΔ*G*_3_ – Φ*q* values are listed in Table 7 for Φ = −14.53 kcal/(mol e), which is the air/water interface potential estimated for the TIP4P model.^36,53^ Due to the possible origin of *A*,^17,32^ Φ represents an important reference value. Indeed, values of *A* obtained at 1 atm for Cs^+^, Br^–^, and I^–^ are very close to this value. This still suggests that *A* mainly is due to water molecules at the boundary, whose orientation could be similar to that found at the air/water interface. However, differences of only a few kcal/mol can likely reflect differences between the boundary and the air/water interface.

Regarding the single ions’ hydration free energy, differences between different experimental data can be related to the air/water interface potential.^6,15^ This depends on the different method used to obtain these data. In Figure 6, we show, for example, the comparison of the Tissandier et al.^11^ and Noyes^10^ data with the Marcus compilation.^1^ Although these data are all absolute hydration free energies, discrepancies are shown in the typical asymmetry between anions and cations. The straight line in the figure (*y* = *x*) represents data with the same value as the Marcus compilation. The other experimental data fall above this line (shadow region) for anions and below this line for cations. According to the distinction made by Lamoureux and Roux^15^ between the *real* and *intrinsic* hydration free energies of a single ion, the Tissandier et al. data should be considered *real* as these should include the air/water interface potential. The same figure reports our FEP results, uncorrected and corrected by *C*_LR_. Interestingly the latter are in between the data of Tissandier et al. and Noyes.

### Extrapolating Errors at Higher Pressures

At higher pressures, we can assume an improvement of FEP results by the addition of – *Aq*, since at 1 atm this yields better agreement with the experimental data of Δ*G*_hyd_^*^ (see Table 7). As an alternative, we propose here to extrapolate errors at higher pressures from errors at 1 atm of the FEP results corrected by *C*_LR_ + *C*_WW_. The extrapolation makes use of the water number density from which we define the corresponding correction,
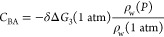
20

In this way, at 1 atm, *C*_BA_ = −δΔ*G*_3_(1 atm), and the estimated value μ* equals Δ*G*_hyd_^*^ of the Marcus compilation.^1^ It is worth noting that also Ashbaugh and Wood^17^ proposed a correction to MPT free energies proportional to ρ_w_. However, in their equation, the proportional constant depends only on the parameters of the water model potential used in simulation, whereas in our case, it depends also on the ion charge. Indeed, at 1 atm, we found a difference in the absolute values of δΔ*G*_3_ between the alkali metal and halide ions (Table 7). It is likely that the correction defined by eq 20 includes also corrections to errors due to assumptions made in the evaluation of corrections *C*_LR_ and *C*_WW_.

### How Corrections Affect the Pressure Profiles of the Chemical Potential of Single Ions and Electrolytes

Here, we compare three sets of results derived from the FEP calculations by the addition of three different corrections. In the first one, *C*_1_ is defined as the sum of *C*_LR_ and *C*_WW_, from which by adding *C*_BA_ we define the second correction, *C*_2_. The third correction, *C*_3_, is instead obtained by subtracting *Aq* from *C*_1_. The correction *C*_BA_ was computed using the number density of TIP4P water^36,45^ (eq 20), while values of *A* were those plotted in Figure 8.

**Figure 9:**
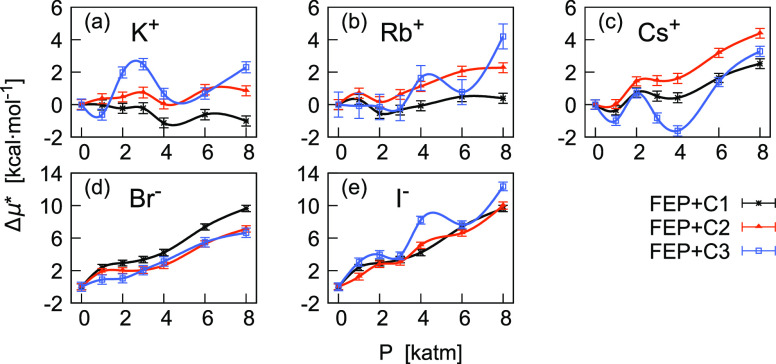
Pressure dependence of Δμ* for single ions, that is the difference between values at *P* with respect to values at 1 atm. In the top panels, results for alkali metal ions, and in the bottom panels, results for halide ions are shown. Comparison between FEP results obtained with three different corrections. All results include the correction *C*_1_ = *C*_LR_ + *C*_WW_, being *C*_2_ = *C*_1_ + *C*_BA_ and *C*_3_ = *C*_1_ – *Aq*.

In Figure 9 we show the corresponding pressure profiles for Δμ*, which is the difference in μ* between pressure *P* and 1 atm. Curves are the cubic splines of the computed points. At any pressure, *C*_2_ – *C*_1_ and *C*_3_ – *C*_1_ have the same sign (positive for cations and negative for anions) but different values and show different behavior with increasing pressure. In particular, the pressure dependence of *C*_BA_ is determined by ρ_w_, and, consequently, the separation of curves relative to *C*_2_ from those relative to *C*_1_ increases with increasing pressure. On the other hand, the trend characterizing pressure dependence of *A* (Figure 8) reflects in curves relative to *C*_3_.

Although *C*_3_ and *C*_2_ are based on unrelated equations, eqs 19 and 20, differences in the corresponding values of Δμ* are in some cases very close within statistical uncertainties. The best agreement is observable for Br^–^ (left bottom panel of Figure 9). For Rb^+^, there is generally a reasonable agreement, even if the cubic splines are qualitatively different. The most significant discrepancies are found for K^+^ at 2000 and 3000 atm, for Cs^+^ at 3000 and 4000 atm, and for I^–^ at 4000 atm.

Although we do not have enough elements to prefer *C*_2_, this should give more reliable results because the correction is based on the study of the errors estimated at 1 atm (eq 20). The most relevant change induced by this correction is observed on Δμ* of alkali metal ions, which after the correction becomes positive in the whole range of pressure. This implies that the LJ component of μ* dominates the pressure effect also in these cases, though not as much as we observed for halide ions.

**Figure 10:**
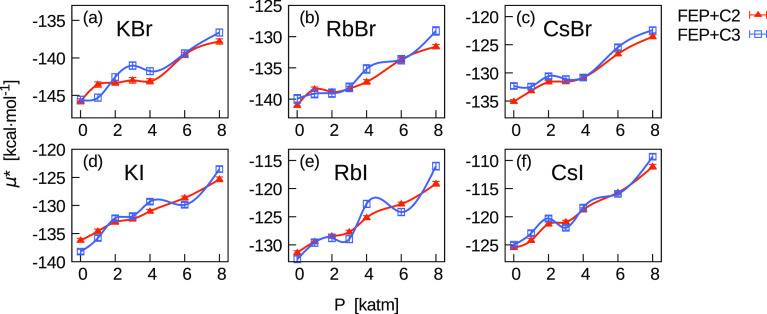
Pressure dependence of μ* for electrolytes expressed as the sum of chemical potentials of single ions. In the top panels, results for bromide, and in the bottom panels, results for iodide electrolytes are shown. Comparison between FEP results obtained with two different corrections (*C*_2_ and *C*_3_). All results include the correction *C*_1_ = *C*_LR_ + *C*_WW_, being *C*_2_ = *C*_1_ + *C*_BA_ and *C*_3_ = *C*_1_ – *Aq*.

Finally, in Figure 10, we show the pressure dependence of the chemical potential of bromide (top panels) and iodide electrolytes (bottom panels). Data were computed as a sum of the chemical potentials of K^+^, Rb^+^, and Cs^+^ with those of Br^–^ and I^–^. For electrolytes, chemical potentials computed using corrections *C*_2_ and *C*_3_ are in good agreement, generally within statistical uncertainties. In particular, this occurs in the whole range of pressures for CsI. Likely effects in *A* not ascribable to the boundary artefact were at least partially eliminated when computing the sum of chemical potentials.

### Pressure Dependence of μ* and Excess Volumes

The pressure dependence of the chemical potential of a solute species can give important information on the excess volume, v*, as this is the partial derivative of μ* with respect to *P* when *T* and the number of all other chemical species in the system are kept constant. For single ions, this is purely a formal definition, and conventions are therefore established, as is usual, for single-ion quantities. In particular, for a variety of ions, the Marcus compilation^1^ provides also experimental data of partial molar volumes, from which v* differ by *kTk*_T_ (1.1 cm^3^/mol at 298.15 K, *k*_T_ being the isothermal compressibility of water). In principle, the pressure dependence of our computed values of μ* can be validated by comparison with these experimental data. However, this comparison assumes a linear pressure dependence of μ*, the reference data being measured at 1 atm.

Linearity has also been assumed when using the so-called slope method^54,55^ to compute partial molar volumes from the simulation data of Δ_hyd_G*. Due to the statistical uncertainties in free energy results, inaccuracy in the second partial derivative seems to blur the distinction between linear and nonlinear dependence. In addition, the pressure effect can be small, and differences in free energies used to estimate the derivative can refer to a very large variation in *P* (5000 bar).^54^ Similar difficulties in the evaluation of excess volumes regard our results, despite statistical uncertainties being within 0.2–0.4 kcal/mol (Table 5).

**Figure 11:**
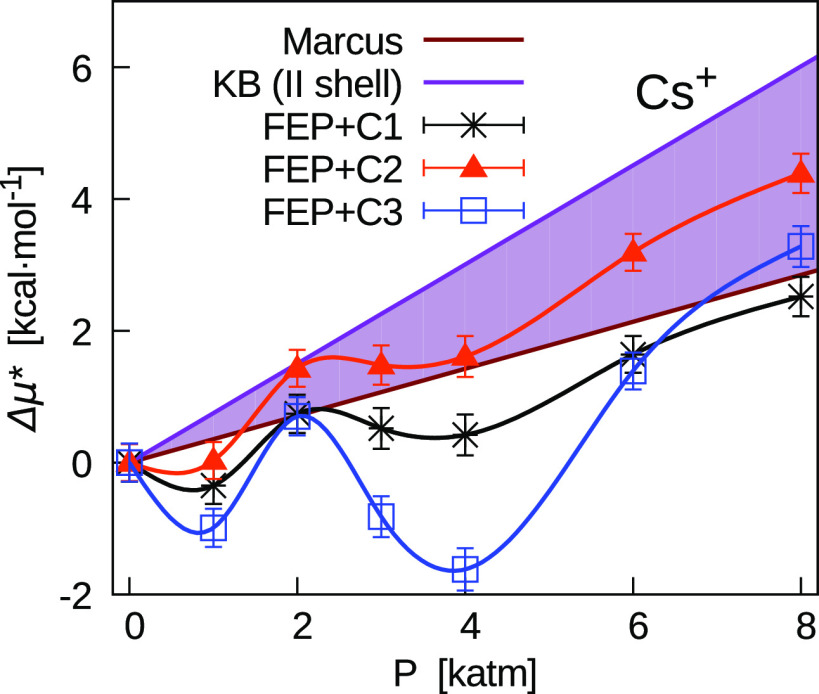
Pressure dependence of Δμ* = μ*(*P*) – μ*(1 atm) for Cs^+^. Comparison between the FEP results obtained with three different corrections (*C*_1_, *C*_2_, and *C*_3_) and linear dependence (Δμ* = (*P* – 1 atm)*v*) based on v* derived from the Marcus compilation.^1^ The colored region is delimited by this straight line and that whose slope is based on KB_II_ determined from simulation rdfs.^3^

**Figure 12:**
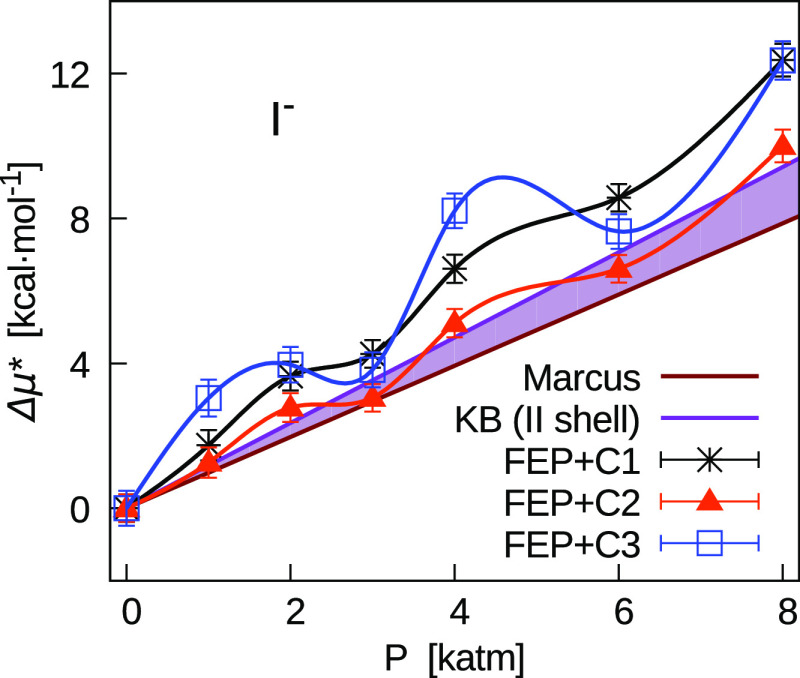
Pressure dependence of Δμ* = μ*(*P*) – μ*(1 atm) for I^–^. Comparison between the FEP results obtained with three different corrections (*C*_1_, *C*_2_, and *C*_3_) and linear dependence (Δμ* = (*P* – 1 atm)*v*) based on v* derived from the Marcus compilation.^1^ The colored region is delimited by this straight line and that whose slope is based on KB_II_ determined from simulation rdfs.^3^

**Figure 13:**
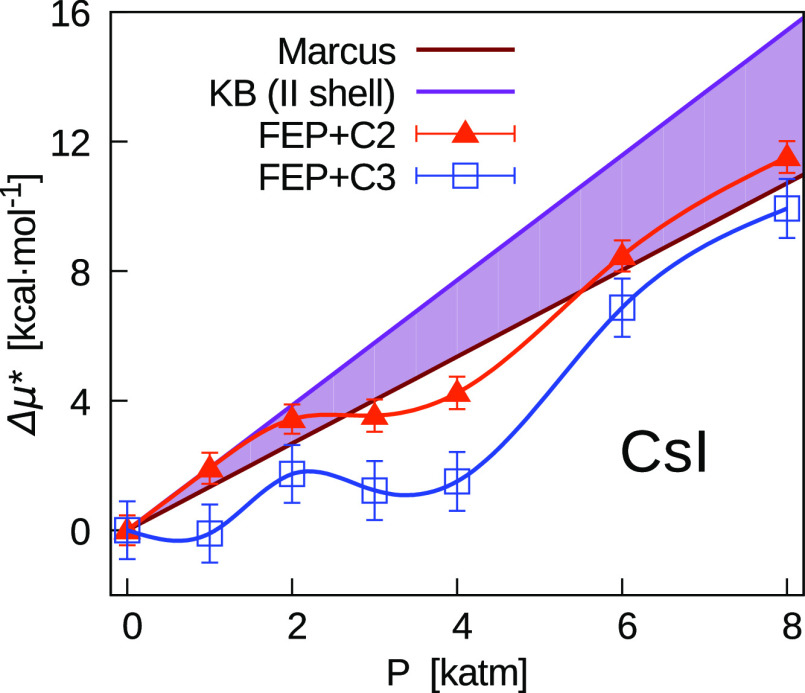
Pressure dependence of Δμ* = μ*(*P*) – μ*(1 atm)for CsI. Comparison between FEP results obtained with three different corrections (*C*_1_, *C*_2_, and *C*_3_) and linear dependence (Δμ* = (*P* – 1 atm)*v*) based on v* derived from Marcus compilation.^1^ The colored region is delimited by this straight line and that whose slope is based on KB_II_ determined from simulation rdfs.^3^

Here, we prefer to compare Δμ* with the straight line based on the excess volumes derived from the Marcus compilation.^1^ As an example, in Figures 11–13 we show this comparison for Cs^+^, I^–^, and CsI, for which we observed the most significant pressure effect. The colored region in the figures corresponds to points in between the two straight lines with slopes equal to v* of the Marcus data^1^ and the Kirkwood–Buff^56^ (KB) integral truncated at the second minimum (KB_II_) of the ion-O rdf obtained in previous simulations^3^ at 1 atm. Thus, KB_II_ contains contributions from the spherical excluded volume and from the two first hydration shells.

KB integrals enable correlation of pressure-induced changes in μ* with structural changes in the hydration shells^3^ and offer a valid method to compute v*. However, due to the long-range action of the ion field, the truncation of the KB integral can be very critical.^57^ We found KB_II_ more positive than v* derived from the Marcus compilation, except for Br^–^ a 1 atm. This suggests that contributions beyond the second shell should be of a negative sign for K^+^, Rb^+^, Cs^+^, and I^–^. A study with a larger box is necessary to verify this inference, particularly at higher pressures. On the other hand, Dahlgren et al.^54^ found a decrease in partial molar volumes when increasing the system size, which lends support to our observation.

While the fact remains that we do not have high-pressure data for v*, it is useful to reflect on some points in order to evaluate the pressure dependence of μ*. The first point is that KB_II_ remains positive at pressures higher than 1 atm. As a consequence, a negative value of v* assumes the dominance of the negative long-range correction. The second point is that from earlier measurements (NaCl in water), it has been concluded that v* of electrolytes mainly increases under increasing pressure.^27^ This suggests that, also at higher pressures, v* has a positive sign for KBr, KI, RbBr, RbI, CsBr, and CsI. Otherwise, it must be assumed that the measured pressure effect may have been distorted in the case of finite concentration.^26,58^

As for the cases illustrated in Figures 11–13, it is worth noting that the statistical uncertainties are significantly smaller than the range of Δμ*. Thus, a clear trend is shown by the data that indicate the possibility of a significant negative slope for the FEP + *C*_3_ results. In particular, this is relevant for Cs^+^ at lower pressures and between 2000 and 4000 atm. This would imply negative v* in disagreement with experimental data at 1 atm. For higher pressures, we can refer to the first point above and conclude that a negative slope of μ* can be possible, but it appears more difficult to justify (see the second point above).

On the contrary, in the majority of ions, values of μ* at 1000 atm of the FEP + *C*_2_ results are close to the Marcus straight line within a few times the statistical uncertainty. This provides a further reason to prefer correction *C*_2_ with respect to correction *C*_3_. In particular, results for halide ions are significantly improved, as shown for I^–^ in Figure 12. As for the whole range of pressure, we generally observe that the FEP + *C*_2_ results lay between the two straight lines.

Nevertheless, for all ions studied in this work, the nonlinear behavior of the interpolating curves presents similar features, which are more or less pronounced depending on the correction. It appears that these were already present in the original FEP results. For example, the curves highlight a change of regime at intermediate values of pressure (Figures 11–13). Although statistical uncertainties prevent being confident on what is suggested by the interpolating curves, the presence of a critical point in μ*(*P*) cannot be excluded a priori. In fact, the lack of information on excess compressibilities and their derivatives with respect to *P* has generally limited the functions used to describe the possible nonlinear behavior.^54,59^

## Summary and Conclusions

We have investigated the pressure effect on the chemical potential of some alkali metal ions and two halide ions, all characterized by excess volumes of positive sign at 1 atm. The increase in the chemical potential under increasing pressure is therefore suggested. This would imply that changes are dominated by the work of cavity formation, according to a simplified decomposition of the chemical potential. FEP calculations combined with simulations allow us to decompose the chemical potential into two terms that can be relevant for modeling free energy contributions at different conditions of pressure. In line with the simplified decomposition, we can expect that changes induced by increasing pressure are dominated by the hydrophobic component obtained by the coupling of LJ ion–water potential. This was observed in the original FEP results for the larger ions we have studied, Cs^+^, Br^–^, and I^–^. While it is clear that the Born formula provides a simplified description of the pressure effect on the hydrophilic component, in particular without changing the cavity radius, attention should be paid to the simulation conditions that affect this component.

For an ion in water, FEP results of μ* obtained by using MPT are subjected to errors that cannot be neglected both when making a comparison at 1 atm with experimental data and when making a prediction at higher pressures. Indeed, the good agreement with experimental data for the difference in the free energy of hydration between ions of the same charge implies some cancellation of these errors. From the comparison at 1 atm, our investigation has shown that these errors can be reasonably managed by adding corrections to the original FEP results. In particular, we propose an original method to compute the correction for the truncation of the Coulomb water–water interactions, *C*_WW_ (eqs 16 and 17). Our method is based on the solution of the Poisson equation applied with and without truncation in the calculation of the matrix **T**, whose inversion leads to the polarization charges defined on the cavity surface. Values of *C*_WW_ are in line with those computed by using more elaborate expressions proposed in the literature.

The addition of *C*_WW_ and *C*_LR_ to the FEP results improves the estimate of the chemical potential of halide ions and of the corresponding electrolytes containing the alkali metal ions. However, after these corrections, errors (δΔ*G*_3_) show the characteristic asymmetry between cations and anions that can be ascribed to boundary artifacts introduced by MPT. A possible correction is computed as – *Aq*, with *A* being the static electrostatic potential, which is obtained from the quadratic fitting of the FEP results of Δ*G*(LJ → LJ + *q*) as a function of the coupled charge (eq 19). Despite the fact that this term might include also other effects, the addition of this correction improves both the chemical potential of single ions and those of electrolytes. The remaining errors can be ascribed to overlapping effects in *A* or to limitations of the continuum model that is used to compute *C*_LR_ and *C*_WW_. The origin of the static electrostatic potential undoubtedly deserves further investigation. In this way, a more accurate correction can be formulated for the boundary artifact. However, the alternative correction based on eq 20 appears to be reasonable, also on the basis of comparison with results derived from excess volumes of the Marcus compilation.^1^

Taking into account corrections for the boundary artifact, we conclude that an increase in pressure determines a higher value of the chemical potential regardless of the charge, even though larger changes are observed for halide ions. Comparing ions of the same charge, the effect is stronger with an increase in the ion size. For all monovalent ions studied in this work, the hydrophobic component has therefore a prominent role on the chemical potential of single ions and the derived electrolytes. As this component corresponds to charging the LJ ion–water potential, this highlights the importance of model potentials in the study of the pressure dependence of the chemical potential of ions in aqueous solutions.

For a simple model defined as the sum of a LJ term plus a Coulomb interaction term with TIP4P water, the parameters of Jensen and Jorgensen^6^ are optimal at 1 atm. Their transferability^60−63^ at higher pressures has been assumed in this work. The validity of this assumption partially depends on the ability of TIP4P to reproduce the properties of liquid water along the isotherm. This is particularly true of density, isothermal compressibility, and dielectric constant, which is relevant in the study of ionic aqueous solutions. Overall, the TIP4P model has a good performance^60,64^ among the rigid nonpolarizable models of water. However, all these models can suffer of limitations^65^ at short intermolecular distances, which can be relevant at high pressures. Some improvements have been suggested in the literature.^64^ In principle, a flexible and polarizable model for water should be better for the study of ionic aqueous solutions^15,62^ at high pressure even if these are computationally more expensive. Moreover, the explicit description of the ion-polarization^15,62^ can also be important, particularly for anions. For Cl^–^ and I^–^, the Drude polarizable ion model^15^ has shown a more favorable free energy of hydration (within 2 kcal/mol) and smaller excluded volumes than the nonpolarizable ion model.^63^ Similar comparison can be used at higher pressures to investigate the transferability of the ion LJ parameters,^6^ which implicitly include the ion-polarization effects.

Finally, TIP4P/ϵ^66^ is a recent water model based on the dielectric constant that can give some improvement of the results without increasing the cost of simulations. Without changing the force field parameters for water–water interactions, some improvement can also be expected by a less simple ion–water model,^67^ which introduces additional uncharged sites on water with terms that include the exponential function and additional power terms of the inverse of the ion-site distance. This function was proposed to fit two-body effective ion–water potentials obtained from ab initio quantum mechanical calculations with a specific method^60,67,68^ to include non-additivity. It has given a good description of the hydration structure at 1 atm and at higher pressures.^67−69^

## Supporting Information Available

Test results on the cutoff setup at different pressures (PDF)
