# Supplementary material for: Hydrophilic Versus Hydrophobic Coupling in the Pressure Dependence of the Chemical Potential of Alkali Metal and Halide Ions in Water
Source: J Phys Chem B. 2022 Nov 3;126(45):9325–38. doi: 10.1021/acs.jpcb.2c02373 (PMC9677433; doi:10.1021/acs.jpcb.2c02373)
Supplement: Supplementary file 1 — jp2c02373_si_001.pdf [file jp2c02373_si_001.pdf]

SUPPORTING INFORMATION

for

”Hydrophilic versus hydrophobic coupling in the pressure dependence of the chemical potential of alkali metal and halide ions in water”

Luca Tonti[*,†] and Franca Maria Floris[*,‡]

†*Department of Chemical Engineering, The University of Manchester, Manchester M13 9PL, UK*

‡ *Dipartimento di Chimica e Chimica Industriale, Università di Pisa,*

Via Giuseppe Moruzzi 13, 56124 Pisa, Italy

E-mail: luca.tonti@manchester.ac.uk; francamaria.floris@unipi.it

Test results on the cutoff setup at different pressures

Here, we consider the effect of a different cutoff setup on the FEP results both uncorrected and corrected by $C_{LR} + C_{WW}$.

We exploit information derived from detailed studies[1] at 1 atm and limit our tests to cutoff radii of 10 and 11.5 Å. According to the literature, the best performance in terms of convergence is reached beyond 9 Å when $R_{WW} = R_{IW}$. This is confirmed by results shown in Table 1, which are relative to $\Delta G(LJ \to LJ + q)$ computed for a cutoff radius of 10 Å. Indeed, their difference with respect to results obtained with a cutoff of 11.5 Å, $\delta\Delta G_{FEP}$, is within the statistical uncertainty for Rb^+ and for I^-. For the smaller ions, K^+ and Br^-, this difference is larger but within three times the statistical uncertainty. At this pressure, the correction term $C_{LR} + C_{WW}$ is -8.2 kcal/mol for $R_{IW} = R_{WW} = 10$ Å and -7.4 kcal/mol for $R_{IW} = R_{WW} = 11.5$ Å, respectively. The difference between these corrections (-0.8 kcal/mol) determines the difference between $\delta\Delta G_{FEP}$ and $\delta\Delta G^{corr}_{FEP}$, which is the difference between corrected results obtained for the two different cutoff radii. For the corrected values the convergence remains generally good but it is less good for K^+.

A less good performance is obtained when different cutoffs are used for water-water and ion-water interactions[1]. By way of example, we show results for $R_{WW} = 10$ Å and $R_{IW} = 11.5$ Å. A complete test was done for I^-, at all values of pressure we studied along the isotherm. Results are shown in Table 2 and in Table 3, for the two contributions $\Delta G(LJ \to LJ + q)$ and $\Delta G(0 \to LJ)$. In order to test convergence, comparison is still made with respect to values relative to the choice $R_{WW} = R_{IW} = 11.5$ Å. Thus, in this test Born's correction (C_{LR}) is the same while C_{WW} is 5.78 kcal/mol and 5.03 kcal/mol at 1 atm respectively for the smaller and the larger R_{WW}. The correction weakly depends on pressure (see Methods section of main text) and the difference between C_{WW} remains practically constant along the isotherm (0.74-0.75 kcal/mol). The variation of $\delta\Delta G_{FEP}$ with pressure is in a quite limited range, with the maximum difference of -1.2 kcal/mol between results at 4000 atm with respect to results at 1 atm. For this setup the corrected FEP results show better convergence, even if $\delta\Delta G^{corr}_{FEP}$ are still significantly negative, as shown in Table 2.

Finally, results relative to the coupling of the LJ component of the ion-water interaction are gener-

ally little affected by different R_{WW}, as shown in Table 3. Indeed, apart for 3000 atm, the values of $\delta\Delta G_{FEP}$ are within two times the statistical uncertainty of the FEP results.

Table 1: FEP results of $\Delta G(LJ \rightarrow LJ + q)$ at 1 atm and 298.15 K obtained using a cutoff of 10 Å for both the ion-water and the water-water interactions. Differences with respect to results obtained using the same kind of setup but with a cutoff of 11.5 Å are reported in the second column ($\delta\Delta G_{FEP}$). The last two columns refer to FEP results corrected by C_{LR} and C_{WW}.

ion	ΔG_{FEP}	$\delta\Delta G_{FEP}$	ΔG^{corr}_{FEP}	$\delta\Delta G^{corr}_{FEP}$
K^+	-76.7(2)	-0.5	-84.9	-1.3
Rb^+	-72.3(2)	0.1	-80.5	-0.7
Br^-	-57.3(2)	0.6	-65.5	-0.2
I^-	-49.2(2)	0.2	-57.4	-0.6

Table 2: FEP results of $\Delta G(LJ \rightarrow LJ + q)$ for I^- at various pressures and 298.15 K obtained using a cutoff of 11.5 Å and of 10 Å for the ion-water and the water-water interactions respectively. Differences with respect to results obtained using a cutoff of 11.5 Å for both interactions are reported in the third column. The last two columns refer to FEP results corrected by C_{LR} and C_{WW}.

P (atm)	ΔG_{FEP}	$\delta\Delta G_{FEP}$	ΔG^{corr}_{FEP}	$\delta\Delta G^{corr}_{FEP}$
1	-52.2(2)	-2.8	-58.8	-2.1
1000	-52.3(2)	-3.2	-59.0	-2.5
2000	-52.2(2)	-3.1	-58.8	-2.4
3000	-51.9(2)	-2.8	-58.5	-2.1
4000	-52.2(2)	-4.0	-58.8	-3.2
6000	-51.6(2)	-2.9	-58.2	-2.2
8000	-51.5(2)	-3.4	-58.1	-2.1

Table 3: FEP results of $\Delta G(0 \rightarrow LJ)$ for I^- at various pressures and 298.15 K obtained using a cutoff of 11.5 Å and of 10 Å for the ion-water and the water-water interactions respectively. Differences with respect to results obtained using a cutoff of 11.5 Å for both interactions are reported in the third column.

P (atm)	ΔG_{FEP}	$\delta\Delta G_{FEP}$
1	1.0(2)	-0.1
1000	2.7(2)	0.2
2000	4.0(2)	-0.4
3000	5.9(2)	0.9
4000	6.8(2)	0.3
6000	8.5(2)	0.4
8000	12.3(2)	0.2

References

(1) Jensen, K. P.; Jorgensen, W. Halide, Ammonium, and Alkali Metal Ion Parameters for Modeling Aqueous Solutions. J. Chem. Theory Comput. **2006**, 2, 1499–1506.
